# Synergistic Neuroprotective Effect of Endogenously-Produced Hydroxytyrosol and Synaptic Vesicle Proteins on Pheochromocytoma Cell Line against Salsolinol

**DOI:** 10.3390/molecules25071715

**Published:** 2020-04-08

**Authors:** Robina Manzoor, Aamir Rasool, Maqbool Ahmed, Ullah Kaleem, Lucienne Nneoma Duru, Hong Ma, Yulin Deng

**Affiliations:** 1 Beijing Key Laboratory for Separation and Analysis in Biomedicine and Pharmaceuticals, School of Life Science, Beijing Institute of Technology, Beijing 100081, China; 3820150009@bit.edu.cn (R.M.); l.duru@bit.edu.cn (L.N.D.); 04656@bit.edu.cn (H.M.); 2 Institute for Synthetic Biosystem, School of Chemistry and Chemical Engineering, Beijing Institute of Technology, Beijing 100081, China; rasool.amir@bit.edu.cn; 3 Institute of Biochemistry, University of Balochistan, Quetta 87300, Pakistan; 4 Department of Tuberculosis, Bolan University of Medical and Health Sciences, Quetta 87300, Pakistan; maqboola93@gmail.com; 5 Department of Microbiology, University of Balochistan, Quetta 87300, Pakistan; drkaleemullah@gmail.com

**Keywords:** Parkinson’s disease, hydroxytyrosol, synapsin-1, septin-5, salsolinol, antioxidants, DOPAL, DOPET

## Abstract

Oxidative stress triggers a lethal cascade, leading to Parkinson’s disease by causing degeneration of dopaminergic neurons. In this study, eight antioxidants were screened for their neuroprotective effect on PC12 cells (pheochromocytoma cell line) under oxidative stress induced by salsolinol (OSibS). Hydroxytyrosol was found to be the strongest neuroprotective agent; it improved viability of PC12 cells by up to 81.69% under OSibS. Afterward, two synaptic vesicle proteins, synapsin-1 and septin-5, were screened for their neuroprotective role; the overexpression of synapsin-1 and the downregulation of septin-5 separately improved the viability of PC12 cells by up to 71.17% and 67.00%, respectively, compared to PC12 cells only treated with salsolinol (PoTwS) under OSibS. Subsequently, the PC12+syn^+^+sep^−^ cell line was constructed and pretreated with 100 µM hydroxytyrosol, which improved its cell viability by up to 99.03% and led to 14.71- and 6.37-fold reductions in the levels of MDA and H_2_O_2_, respectively, and 6.8-, 12.97-, 10.57-, and 7.57-fold increases in the activity of catalase, glutathione reductase, superoxide dismutase, and glutathione peroxidase, respectively, compared to PoTwS under OSibS. Finally, alcohol dehydrogenase-6 from *Saccharomyces cerevisiae* was expressed in PC12+syn^+^+sep^−^ cells to convert 3,4-dihydroxyphenylacetaldehyde (an endogenous neurotoxin) into hydroxytyrosol. The PC12+syn^+^+sep^−^+ADH6^+^ cell line also led to 22.38- and 12.33-fold decreases in the production of MDA and H_2_O_2_, respectively, and 7.15-, 13.93-, 12.08-, and 8.11-fold improvements in the activity of catalase, glutathione reductase, superoxide dismutase, and glutathione peroxidase, respectively, compared to PoTwS under OSibS. Herein, we report the endogenous production of a powerful antioxidant, hydroxytyrosol, from 3,4-dihydroxyphenylacetaldehyde, and evaluate its synergistic neuroprotective effect, along with synapsin-1 and septin-5, on PC12 cells under OSibS.

## 1. Introduction

Parkinson’s disease (PD) is the second most prevalent neurological syndrome; it inflicts a heavy financial burden on the global health system every year [1,2,3,4]. The number of PD cases will increase from 4.1 to 8.7 million by 2030 in the most populous countries of the world [5]. PD is an age-related disease which is characterized by motor dysfunction, i.e., rigidity, tremor, resting, and gait difficulty, and nonmotor dysfunction, e.g., depression, anxiety, cognitive dysfunction, and olfactory dysfunction [6]. PD is caused by a progressive loss of dopaminergic (DAergic) neurons and the formation of Lewy bodies of α-synculein [6,7]. The currently approved drugs used for treatment of PD produce different side effects, and even fail to stop the progression of disease [8,9,10]; this is due to limited knowledge regarding the etiopathology of PD. However, several studies have reported that oxidative stress (OS) initiates neuroinflammation, lysosomal dysfunction, mitochondrial dysfunction, and synaptic dysfunction, which consequently results in the onset of PD [11,12].

Dyshomeostasis between the generation of reactive oxygen species (ROS) and their elimination by the native antioxidant defense system is the main source of oxidative stress in DAergic neurons [13]. The decreased activity of superoxide dismutase, catalase, and glutathione oxidase/reductase has been attributed to the accumulation of ROS in the substantia nigra pars compacta (SNpc) of PD patients [13,14,15]. ROS catalyze the lipid peroxidation of polyunsaturated fatty acids and produce highly reactive electrophilic aldehydes in the brain [16,17]. Oxidative stress activates the microglial cells through neuroinflammation and causes nigral cell death [18,19,20]. The excessive accumulation of ROS triggers the process of mitochondrial dysfunction, which releases H_2_O_2_ and superoxide radicals in the cytoplasm of DAergic neurons [21,22]. The cytosolic accumulation of ROS in DAergic neurons also promotes the formation of aggregates of α-synuclein, which subsequently play a critical role in synaptic and mitochondrial dysfunction [23,24,25].

Optimum densities of synapses are critical for the regeneration of synaptic vesicles and the efficient packaging of dopamine. Earlier studies have already reported a mutation in the DNAJC6 (auxilin), SYNJ1 (synaptojanin 1), and SH3GL2 (endophilin A1) genes by ROS, which subsequently results in synaptic dysfunction and PD [24,26]. The Deng group also reported downregulation of 22 synaptic vesicle proteins, including synapsin-1 and septin-5, in a rat model lesioned by 6-OHDA [27]. The synapsins family is comprised of ˃10% of synaptic vesicle proteins in mammals, which plays a crucial role in the regulation of neurotransmission and synaptogenesis [28]. The readily releasable pool of synaptic vesicles was reduced at the central inhibitory synapses in mice lacking synapsin-1 [29]. Septins serve as scaffolds for a diverse set of molecules and play a vital role in the regulation of exocytosis [30]. Septin-5 is a substrate of parkin and accumulates in the brain due to the loss of parkin [31,32,33,34]. Its acute overload can be inhibitory to exocytosis and can inflict a neurotoxic effect [34,35,36].

The overexpression of heat shock proteins and metalloproteinase TIMP-1 has been shown to demonstrate neuroprotective effects in transgenic mice [37,38]. The overexpression of *bcl-2* blocked the release of cytochrome c from the mitochondria and protected the nigral cells [39]. The overexpression of pleiotrophin also protected the nigrostriatal system, striatum, and SNpc from 6-OHDA toxicity [40]. 

Polyphenols are secondary metabolites produced by plants for their defense, and survive in a hostile milieu [41,42]. They are mainly classified into phenolic acids, flavonoids, stilbenes, and lignans [41,43]. Phenolic acids are nutraceutical compounds that are produced by various plants in different quantities, including cinnamic acid and p-Coumaric acid [41,43]. Flavonoids are the largest family of polyphenols produced by plants [44,45,46], which act as a shield against toxins and help to repair damaged cells [47,48]. Hydroxytyrosol (3,4-dihydroxyphenylethanol; DOPET), a major phenolic alcohol, is found in olive oil and red wine and demonstrates several nutraceutical and pharmaceutical properties [49,50,51,52]. Phenolic compounds display antioxidant activity, and therefore, can be used as neuroprotective agents to prevent neurodegenerative diseases, including PD [53,54,55].

Taken together, herein, a strategy was devised to eliminate oxidative stress and to disrupt the vicious cycle leading to neuroinflammation, synaptic dysfunction, mitochondrial dysfunction, and lysosomal dysfunction in DAergic neurons. For this purpose, firstly, eight antioxidants, i.e., liquiritin, liquiritigenin, isoliquiritigenin, naringenin, hydroxytyrosol, p-Coumaric acid, cinnamic acid, and tyrosol [56,57,58,59,60,61,62], were screened for their strongest neuroprotective effect under OSibS. Then, the neuroprotective role of synaptic vesicle proteins, such as synapsin-1 and septin-5, involved in the exocytosis and endocytosis of synaptic vesicles was determined under OSibS. Afterward, the combined neuroprotective effect of synaptic vesicle proteins and hydroxytyrosol (the strongest antioxidant of this study) on PC12 cells was determined under OSibS.

Through a literature survey, we discovered that alcohol dehydrogenase-6 *(ADH6)* from *Saccharomyces cerevisiae* catalyzes the conversion of 3,4-dihydroxyphenylacetaldehyde (DOPAL) into hydroxytyrosol with high efficiency [63,64]. DOPAL, an endogenous neurotoxin, is produced from the oxidative deamination of dopamine catalyzed by monoamine oxidases [65]. Therefore, alcohol dehydrogenase-6 was overexpressed in the cell line constructed by overexpressing synapsin-1 and downregulating the septin-5 for the endogenous production of hydroxytyrosol to strengthen the native antioxidant defense system of PC12 cells.

## 2. Results and Discussion

### 2.1. Screening Powerful Antioxidants and Neuroprotective Agents

**Figure 1:**
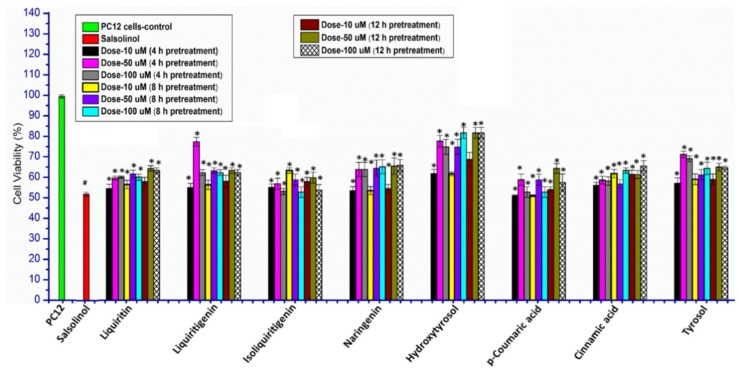
Comparative antioxidant activity of flavonoids measured via MTS assay. The comparative dose and time course-dependent antioxidant activity of liquiritin, liquiritigenin, isoliquiritigenin, naringenin, hydroxytyrosol, p-Coumaric acid, cinnamic acid, and tyrosol were determined for PC12 cells. Hydroxytyrosol demonstrated the strongest antioxidant activity compared to the other antioxidants. Data are mean and S.E. values from three independent experiments (*n* = 3). *p* < 0.01, relative to PC12 (control cells). * *p* < 0.05 and ** *p* < 0.01, relative to the cells treated only with salsolinol.

Phenolic compounds are widely distributed in the plant kingdom and perform a variety of functions—in particular, antioxidation reactions [66,67]. The strength of an antioxidant depends upon the number and type of substitutions on the phenolic ring [66,67]. Therefore, the antioxidant activity of different phenolic compounds was determined to find out the most powerful antioxidant agent. For this purpose, the antioxidant activity of liquiritin, liquiritigenin, isoliquiritigenin, naringenin, hydroxytyrosol, p-Coumaric acid, cinnamic acid, and tyrosol was determined under OSibS. The pretreatment of PC12 cells with liquiritin, liquiritigenin, isoliquiritigenin, naringenin, hydroxytyrosol, p-Coumaric acid, cinnamic acid, and tyrosol enhanced the viability of PC12 cells to different levels compared to PoTwS cells under OSibS. The optimal dose and time of pretreatment of the PC12 cells for each compound were determined by using different doses, i.e., 10, 50, and 100 µM, and times, i.e., 4, 8, and 12 h (Figure 1). PC12 cells pretreated with liquiritin, liquiritigenin, isoliquiritigenin, naringenin, hydroxytyrosol, p-Coumaric acid, cinnamic acid, and tyrosol at doses of 50, 50, 10, 100, 100, 50, 100, and 50 µM for 12, 4, 8, 12, 8, 12, 12, and 4 h helped to recover the viability of PC12 cells by up to 64.28%, 77.36%, 63.28%, 65.1%, 81.69%, 64.29%, 65.51%, and 71.17%, respectively (Figure 1). The results of the MTS assay {(3-(4,5-dimethylthiazol-2-yl)-5-(3-carboxymethoxyphenyl)-2-(4-sulfophenyl)-2H-tetrazolium)} demonstrated that hydroxytyrosol is the strongest antioxidant among the eight antioxidants tested in this study.

The production of malondialdehyde (MDA) and hydrogen peroxide (H_2_O_2_) indicates the level of oxidative stress on neuronal cells and the strength of the endogenous antioxidant defense system (EADA). Malondialdehyde (MDA is produced from the peroxidation of polyunsaturated fatty acid (PUFA), which is catalyzed by ROS [68]. Hydrogen peroxide (H_2_O_2_) is produced in neuronal cells during different reactions and plays a prominent role in the formation of α-synuclein aggregates [69]. The pretreatment of PC12 cells with hydroxytyrosol strongly relieved the oxidative stress induced by salsolinol and mitigated the production of MDA and H_2_O_2_ compared with the other antioxidants tested in this study (unpublished data). 

### 2.2. Synergistic Neuroprotective Effect of Synaptic Vesicle Proteins and Hydroxytyrosol

**Figure 2:**
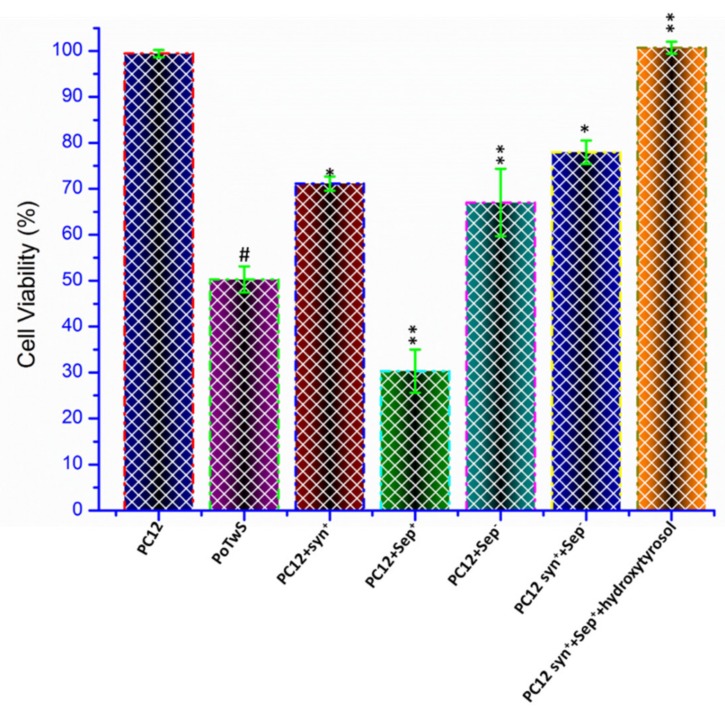
Neuroprotective effect of synaptic vesicle proteins and hydroxytyrosol. The neuroprotective effect of synapsin-1, septin-5, and hydroxytyrosol (12 h, 100 µM) was determined for all cell lines of this study. However, cell line PC12+syn^+^+sep^−^+hydroxytyrosol, which was pretreated with hydroxytyrosol, displayed highest cell viability compared to the other cell lines. The description of each cell line is given in Supplementary Table S3. Data are mean and S.E. values from three independent experiments (*n* = 3). # *p* < 0.01, relative to PC12 (control cells). * *p* < 0.05 and ** *p* < 0.01, relative to the cells treated only with salsolinol. (+) represents the overexpression of a gene, while (–) represents the downregulation of a gene.

Synaptic vesicles are lipid bilayer structures and are used for the storage of neurotransmitters. The synaptic vesicle proteins are classified into two categories based on their function: (1) Transport proteins, and (2) trafficking proteins [70,71]. The synapsin family plays a crucial role in the regulation of neurotransmission and synaptogenesis [28]; meanwhile, septins serve as scaffolds for a diverse set of molecules and play a vital role in regulating the exocytosis of synaptic vesicles [30]. For the determination of individual and synergistic neuroprotective effects of synaptic vesicle proteins and hydroxytyrosol, cell lines such as PC12+syn^+^ (overexpressed the synapsin-1), PC12+sep^+^ (overexpressed the septin-5), PC12+sep^−^ (downregulated the septin-5), PC12+syn^+^+sep^−^ (overexpressed the synapsin-1+ downregulated the septin-5) were constructed, and cell lines pretreated with hydroxytyrosol were named PC12+hydroxytyrosol and PC12+syn^+^+sep^−^+hydroxytyrosol. PoTwS cells (PC12 cells only treated with salsolinol) were used for the measurement of the neuroprotective effect of each treatment performed in this study. The overexpression of synapsin-1 improved the viability of PC12+syn^+^ cells by up to 71.17%, while the overexpression of septin-5 reduced the viability of PC12+sep^+^ cells by up to 30.33%, respectively, compared to PoTwS cells under OSibS (Figure 2). This demonstrates that the overexpression of synapsin-1 had a cytoprotective effect, while septin-5 inflicted a neurotoxic effect on PC12 cells under OSibS.

Therefore, the expression of septin-5 was downregulated using siRNA, which consequently enhanced the cell viability of PC12+sep^−^ by 67% compared to PoTwS cells under OSibS (Figure 2). The viability of PC12+syn^+^+sep^−^ cells was enhanced by up to 77.99% compared to PoTwS cells under OSibS due to the overexpression of synapsin-1 and the downregulation of septin-5, which confirmed the neuroprotective role of these synaptic vesicle proteins (Figure 2). The mechanism of the neuroprotective effect of the aforementioned synaptic vesicle proteins was determined through evaluating their regulatory effect on the exocytosis and endocytosis of the synaptic vesicles of DAergic neurons (unpublished data). The expression and downregulation of the respective synaptic vesicle proteins was confirmed via Western blotting (Supplementary Figure S3a,b,c).

In this study, only the PC12+syn^+^+sep^−^ cell line was pretreated with hydroxytyrosol to determine the synergistic neuroprotective effect of hydroxytyrosol and synaptic vesicle proteins under OSibS. The PC12+syn^+^+sep^−^+hydroxytyrosol cells displayed an increase in cell viability by up to 99.03% compared to PoTwS cells under OSibS (Figure 2). The results of the MTS assay indicated that the pretreatment of hydroxytyrosol, the overexpression of synapsin-1, and the downregulation of septin-5 complemented each other and exerted a strong neuroprotective effect against salsolinol-induced oxidative stress (OSibS) (Figure 2). 

**Figure 3:**
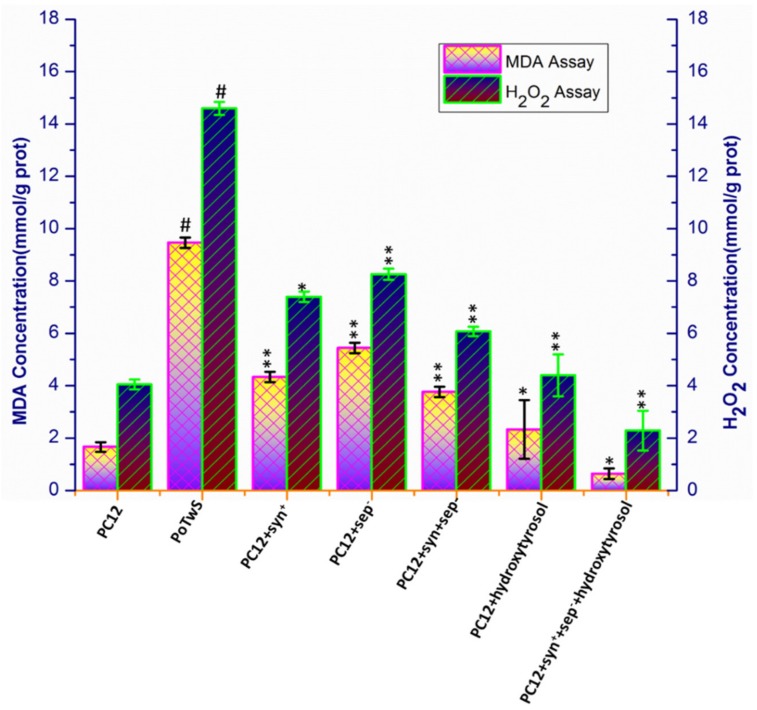
Determination of MDA and H_2_O_2_ levels. The levels of MDA and H_2_O_2_ were increased in PoTwS cells compared to PC12 cells (without any treatment). The PC12+syn^+^+sep^−^+hydroxytyrosol cell line displayed the lowest levels of MDA and H_2_O_2_ compared to PoTwS cells. The PC12+hydroxytyrosol and PC12+syn^+^+sep^−^+hydroxytyrosol cell lines were pretreated with 100 µM of hydroxytyrosol for 12 h. The description of each cell line is given in the Supplementary Table S3. Data are mean and S.E. values from three independent experiments (*n* = 3). # *p* < 0.01, relative to PC12 (control cells). * *p* < 0.05 and ** *p* < 0.01, relative to the cells treated only with salsolinol.

The neuroprotective effective of synaptic vesicle proteins and hydroxytyrosol was further confirmed via measuring the MDA and H_2_O_2_ levels in the aforementioned cell lines. The induction of oxidative stress in the PoTwS cells by salsolinol led to 5.69- and 3.64-fold increases in the accumulation of MDA and H_2_O_2_, respectively, compared to PC12 cells (wild-type cells) (Figure 3). Similarly, the levels of MDA and H_2_O_2_ production in the constructed cell lines, i.e., PC12+syn^+^, PC12+sep^−^, PC12+syn^+^+sep^−^, PC12+hydroxytyrosol, and PC12+syn^+^+sep^−^+hydroxytyrosol also showed 2.19-, 1.74-, 2.52-, 4.06-, and 14.78-fold, and 1.97-, 1.77-, 2.40-, 3.32-, and 6.37-fold, decreases, respectively, compared to PoTwS cells under OSibS (Figure 3). The results of the MDA and H_2_O_2_ assays of the PC12+syn^+^+sep^−^+hydroxytyrosol cell line indicated that synaptic vesicle proteins and hydroxytyrosol exerted a strong synergistic neuroprotective effect against OSibS (Figure 3). 

### 2.3. Effect of Hydroxytyrosol on the Endogenous Antioxidant Defense System of PC12 Cells

Several studies have stressed that OS is produced due to the imbalance between the production of free radicals and the activity of the EADS, which subsequently initiates a vicious cascade, ultimately leading to PD [72,73].

**Figure 4:**
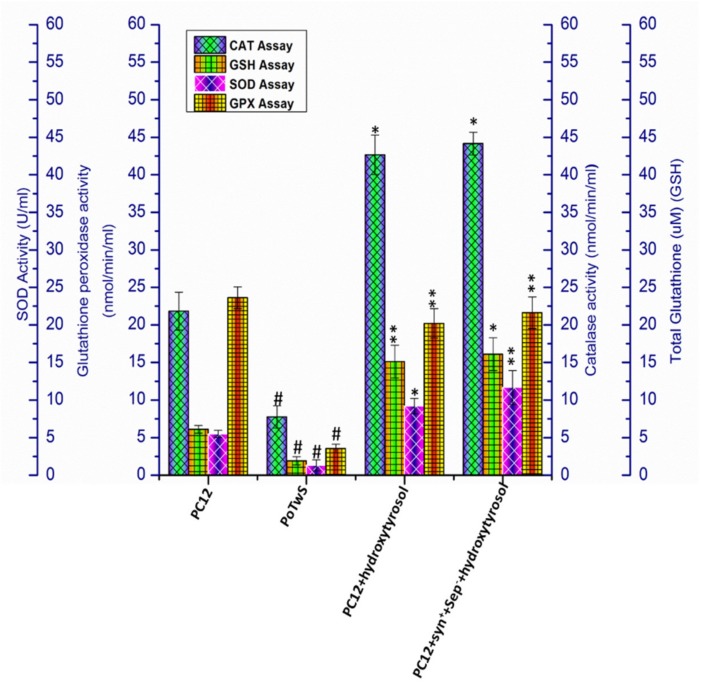
Analysis of activity of catalase (CAT), glutathione reductase (GSH), superoxide dismutase (SOD), and glutathione peroxidase (GPx). The activity of CAT, GSH, SOD, and GPx was decreased in PoTwS cells compared to PC12 cells. Meanwhile, the PC12+syn^+^+sep^−^+hydroxytyrosol cells line displayed the highest CAT and GSH activity compared to PoTwS cells. The description of each cell line is given in Supplementary Table S3. Data are mean and S.E. values from three independent experiments (*n* = 3). # *p* < 0.01, relative to PC12 (control cells). * *p* < 0.05 and ** *p* < 0.01, relative to the cells treated only with salsolinol.

Therefore, the effect of hydroxytyrosol pretreatment on the activity of the EADS of the PC12+hydroxytyrosol and PC12+syn^+^+sep^−^+hydroxytyrosol cell line was determined by performing catalase (CAT), glutathione reductase (GSH), superoxide dismutase (SOD), and glutathione peroxidase (GPx) activity assays. The results demonstrate that the activity of CAT, GSH, SOD, and GPx was decreased 2.81-, 3.20-, 4.14-, and 6.64-fold, respectively, in PoTwS cells compared to PC12 cells under OSibS (Figure 4). However, the catalytic activity of CAT, GSH, SOD, and GPx showed 5.49-, 7.91-, 6.94-, and 5.67-fold increases, respectively, in PC12+hydroxytyrosol cells compared to PoTwS cells under OSibS (Figure 4). On the other hand, the catalytic activity of CAT, GSH, SOD, and GPx was not significantly increased in PC12+syn^+^+sep^−^+hydroxytyrosol cells compared with PC12+hydroxytyrosol cells, indicating that synaptic vesicle proteins do not regulate the activity of the aforementioned enzymes.

The results of the biochemical assays of the PC12+hydroxytyrosol and PC12+syn^+^+sep^−^+hydroxytyrosol cell lines demonstrate that hydroxytyrosol regulates the EADS, which is evident from the increased catalytic activity of CAT, GSH, SOD, and GPx in these cell lines (Figure 4).

### 2.4. Transcriptional Analysis of the Endogenous Antioxidant Defense System

**Figure 5:**
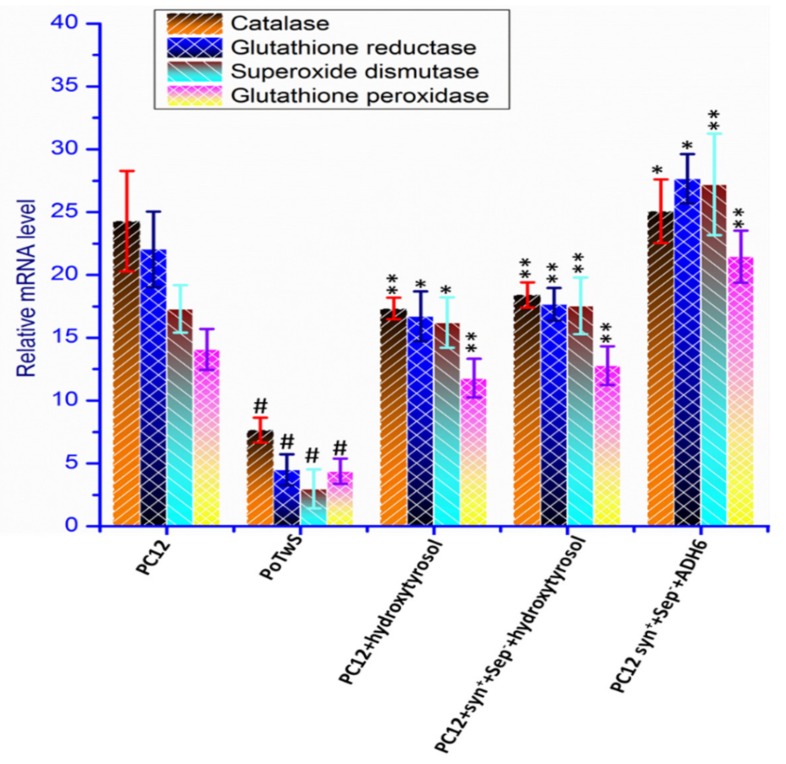
Comparative qPCR analysis. The transcription level of CAT, GSH, SOD, and GPx was determined in PC12 cells, PoTwS cells, and the PC12+hydroxytyrosol, PC12+syn^+^+sep^−^+hydroxytyrosol, and PC12+syn^+^+sep^−^+ADH6 cell lines. The maximum improvement in the transcription level of CAT, GSH, SOD, and GPx was observed in the PC12+syn^+^+sep^−^+ADH6 cell line compared to PoTwS cells. The description of each cell line is given in Supplementary Table S3. Data are mean and S.E. values from three independent experiments (*n* = 3). # *p* < 0.01, relative to PC12 (control cells). * *p* < 0.05 and ** *p* < 0.01, relative to the cells treated only with salsolinol.

Many lines of evidence have shown that the neuroprotective agents, for example, quercetin and epicatechin, positively regulate the expression of CAT, GSH, SOD, and GPx of the EADS at the transcriptional level [74,75]. Therefore, effect of hydroxytyrosol pretreatment on the transcription of CAT, GSH, SOD, and GPx of PC12+hydroxytyrosol and PC12+syn^+^+sep^−^+hydroxytyrosol cells was also evaluated through quantitative PCR (qPCR). The results of the qPCR analysis of PC12+hydroxytyrosol cells displayed 3.19-, 4.82-, 8.27-, and 4.367-fold increases in the transcription levels of CAT, GSH, SOD, and GPx, respectively, compared to PoTwS cells under OSibS (Figure 5). On the other hand, the transcription of CAT, GSH, SOD, and GPx was not significantly increased in PC12+syn^+^+sep^−^+hydroxytyrosol cells compared to PC12+hydroxytyrosol cells, indicating that synaptic vesicle proteins also do not regulate the transcription of the aforementioned enzymes.

The results of the qPCR analysis also indicated that hydroxytyrosol modulated the transcription of CAT, GSH, SOD, and GPx, thereby increasing their activity compared to PoTwS cells under OSibS. The cell lines expressing only synaptic vesicle proteins did not display any stimulatory effect on the transcription and activity of CAT, GSH, SOD, or GPx (unpublished data). The synaptic vesicle proteins and the hydroxytyrosol had a synergistic neuroprotective effect on PC12 cells, perhaps via the activation of different pathways (Figure 2 and Figure 3).

### 2.5. Cytosolic Level of Dopamine and Its Metabolites

A part of dopamine is stored in the synaptic vesicles, while the remainder is degraded into different metabolites by catechol-O-methyl transferase and monoamine oxidases after its influx in the DAergic neurons by the dopamine (DA) transporter [76]. Synaptic vesicle proteins play a crucial role in the regulation of exocytosis and endocytosis of synaptic vesicles. On the other hand, antioxidants such as hydroxytyrosol modulate the activity of the EADS; therefore, both molecules affect the cytosolic contents of DA and its metabolites, either directly or indirectly. Hence, the effect of synapsin-1 overexpression, septin-5 downregulation, and hydroxytyrosol pretreatment was determined on the cytosolic contents of DA and its metabolites (DOPAL and DOPAC) of PC12, PoTwS, PC12+syn^+^, PC12+sep^−^, PC12+syn^+^+sep^−^, PC12+hydroxytyrosol, and PC12+syn^+^+sep^−^+hydroxytyrosol cells using HPLC.

**Figure 6:**
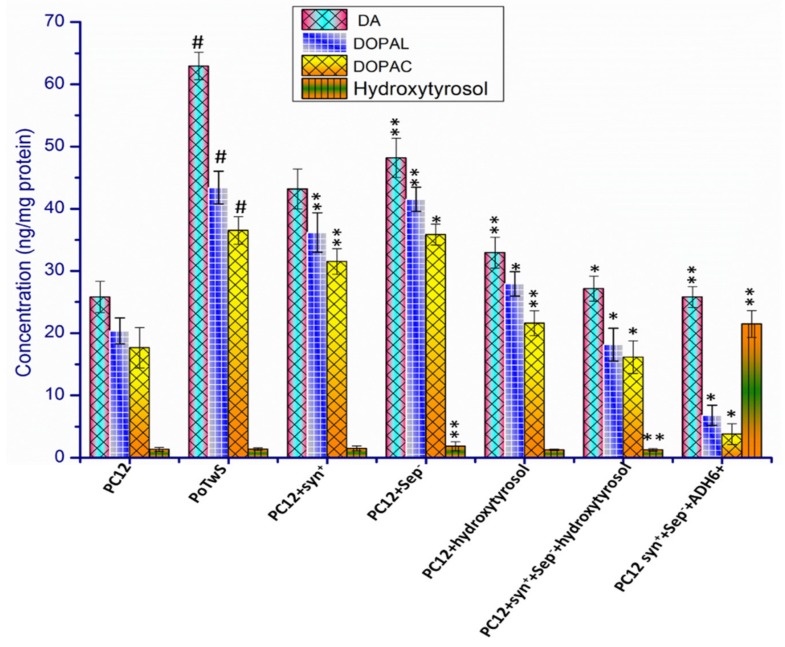
HPLC analysis of dopamine (DA) and its metabolites. The cytoplasmic accumulation of DA, DOPAL, and DOPAC was determined in PC12, PoTwS, PC12+syn^+^, PC12+sep^−^, PC12+hydroxytyrosol, PC12+syn^+^+sep^−^+hydroxytyrosol, and PC12+syn^+^+sep^−^+ADH^+^ cells. PC12+syn^+^+sep^−^+ADH^+^ cells displayed the lowest level of DA, DOPAL, and DOPAC production compared to the other cell lines. The description of each cell line is given in Supplementary Table S3. Data are mean and S.E. values from three independent experiments (*n* = 3). # *p* < 0.05, relative to control cells (PC12 cells). * *p* < 0.05 and ** *p* < 0.01, relative to the cells treated only with salsolinol.

The HPLC analysis results showed an increase in the cytosolic content of DA, DOPAL, and DOPAC in PoTwS cells by 2.43-, 2.13-, and 2.07-fold compared to PC12 cells under OSibS (Figure 6). The cytoplasmic contents of DA, DOPAL, and DOPAC were 1.45-, 1.19-, and 1.16-fold; 1.31-, 1.19-, and 1.16-fold; 1.31-, 1.05-, and 1.02-fold; 1.91-, 2.39-, 2.26-fold; and 2.44-, 6.37-, and 9.58-fold lower in the PC12+syn^+^, PC12+sep^−^, PC12+hydroxytyrosol, and PC12+syn^+^+sep^−^+hydroxytyrosol cell lines, respectively, compared to PoTwS cells under OSibS (Figure 6).

The cytosolic contents of DA, DOPAL, and DOPAC in the PC12+syn^+^+sep^−^+hydroxytyrosol cell line were the lowest compared with the aforementioned cell lines, which indicated that the synaptic vesicle proteins and hydroxytyrosol synergistically protected the PC12 cells from the accumulation of DA and its metabolites. This is perhaps because, on the one hand, hydroxytyrsosol enhanced the activity of the antioxidant defense system, which relieved the oxidative stress on the PC12 cells, and, on the other hand, the synaptic vesicle proteins upregulated the exocytosis and endocytosis of the synaptic vesicles, reducing the exvesicle availability of DA for the production of DOPAL and DOPAC. The production of DA, DOPAL, and DOPAC in all cell lines was detected and confirmed by comparing the spectra of their authentic standard with the samples (Supplementary Figure S4).

### 2.6. Endogenous Production of Hydroxytyrosol from DOPAL 

**Figure 7:**
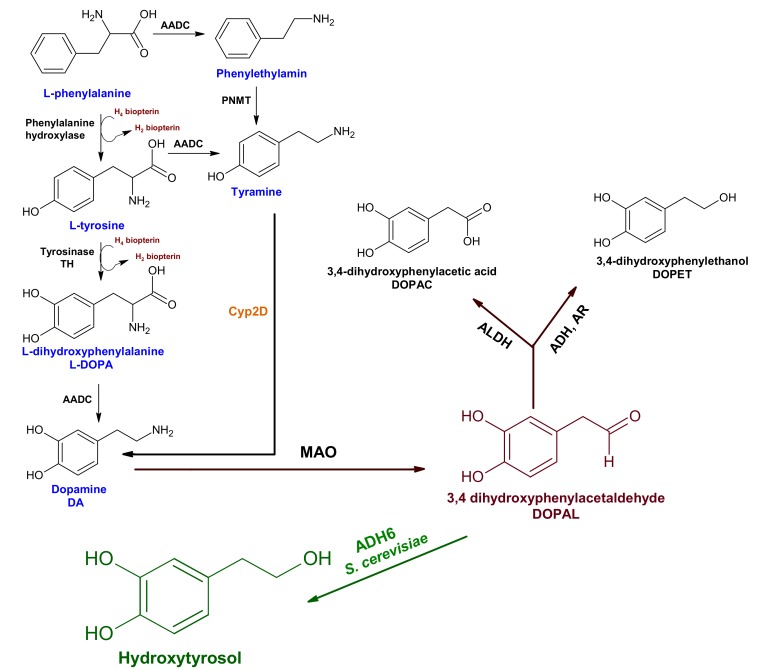
Proposed pathway for the endogenous production of hydroxytyrosol from DOPAL in PC12 cells. In eukaryotes, DA is synthesized via the classical and nonclassical pathways. In the classical pathway, phenylalanine is hydroxylated into tyrosine by phenylalanine hydroxylase and converted into L-DOPA by the action of tyrosinase. Aromatic amino acid decarboxylase (AADC) catalyzes the decarboxylation of L-DOPA into DA. The nonclassical pathway of DA biosynthesis starts with L-phenylalanine decarboxylation into tyramine by AADC, and CYP2D catalyzes the oxidation of tyramine into DA. MAO catalyzes the degradation of DA into DOPAL (an endogenous neurotoxin), which is further degraded into DOPAC (major product) and DOPET (minor product) (written in black). ADH (alcohol dehydrogenase), AR (aldehyde reductase), and ALDH (aldehyde dehydrogenase). In this study, we introduced *ADH6* (*S. cerevisiae*) for the efficient conversion of DOPAL into the hydroxytyrosol and relief of OS (written in green).

Hydroxytyrosol (3,4-dihydroxyphenylethanol; DOPET) containing secoiridoid derivatives has demonstrated the most significant biological activity, such as reduced chronic inflammation and oxidative damage, and acts as an antiproliferative agent [52,77,78,79]. This evidence convinced the European Union to recommend that olive oil polyphenols should contain 250 ppm of hydroxytyrosol and its derivatives [80]. DOPAL is a neurotoxic metabolite of DA, which causes the modification of functional residues of proteins, instigates the aggregation of proteins, and induces OS, leading to the PD. DOPAL has been implicated in the formation of α-synculein aggregates and synaptic dysfunction [81]. The production of DOPAL in DAergic neurons reaches the pathological level only when leakage of dopamine from synaptic vesicles is increased, and the expression of MAO also increases due to OS [81]. The physiological level of DOPAL in DAergic neurons is about 2–3 μM, while the pathological level is ˃6 μM [82]. Generally, DOPAL is catabolized into DOPAC (major product) and DOPET (minor product), perhaps due to the lower catalytic activity of ADH/AR (alcohol dehydrogenase/aldehyde reductase) compared to ALDH (aldehyde dehydrogenase) (Figure 7).

**Figure 8:**
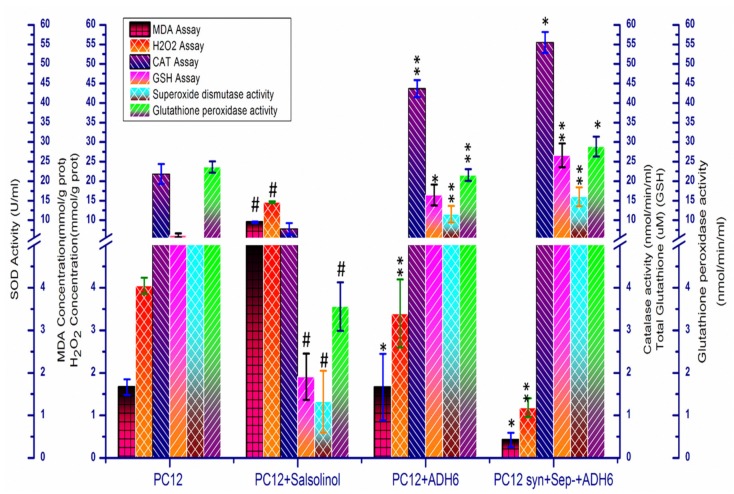
MDA assay, H_2_O_2_ assay, CAT assay, GSH assay, superoxide dismutase activity assay, and glutathione peroxidase activity assay of the PC12+syn^+^+sep^−^+ADH6^+^ cell line. The levels of MDA and H_2_O_2_ were decreased in PC12+syn^+^+sep^−^+ADH6^+^ cells compared to PoTwS cells. The activity of catalase and glutathione reductase activity, superoxide dismutase activity, and glutathione peroxidase was increased in the PC12-ADH6^+^ and PC12+syn^+^+sep^−^+ADH6^+^ cell lines compared to PoTwS cells. Error bars represent the standard deviation of biological triplicates. The description of each cell line is given in Supplementary Table S3). Data are mean and S.E. values from three independent experiments (*n* = 3). # *p* < 0.05, relative to control cells (PC12 cells). * *p* < 0.05 and ** *p* < 0.01, relative to the cells treated only with salsolinol.

Keeping in mind the strongest neuroprotective effect of hydroxyltyrosol, *ADH6* gene from *S. cerevisiae* was overexpressed in the PC12 and PC12+syn^+^+sep^−^ cell lines to endogenously overproduce hydroxytyrosol from DOPAL and to strengthen the EADS of these cell lines. *ADH6* has been previously reported for its efficient catalysis of 3,4-dihydroxyphenylacetaldehyde (DOPAL) into hydroxytyrosol [63,64]. Other studies have also reported the heterologous production of hydroxytyrosol from tyrosol in different bacterial species, such as *Pseudomonas aeruginosa, Serratia marcescens, Escherichia coli,* and *Micrococcus luteus* [83]. This study, for the first time, reports the endogenous overproduction of hydroxyltyrosol to counteract OS and to strengthen the EADS of PC12 cells. The endogenous production of hydroxytyrosol led to 22.38- and 12.33-fold reductions in MDA and H_2_O_2_ production, respectively, in the PC12+syn^+^+sep^−^+ADH6^+^ cell line, compared to PoTwS under OSibS (Figure 8).

The activity of CAT, GSH, SOD, and GPx was increased 7.15-, 13.93-, 12.08-, and 8.12-fold, respectively, in the PC12+syn^+^+sep^−^+ADH6^+^ cell line, compared to PoTwS cells under OSibS (Figure 8). The transcription of CAT, GSH, SOD, and GPx was also enhanced in the PC12+syn^+^+sep^−^+ADH6^+^ cell line due to the endogenous production of hydroxytyrosol (Figure 5). The PC12+syn^+^+sep^−^+ADH6^+^ cells produced 21.47 ng/mg protein hydroxytyrosol; therefore, it accumulated 2.43-, 6.37-, and 9.58-fold lower levels of DA, DOPAL, and DOPAC, respectively, in the cytosol compared to PoTwS cells (Figure 6). The endogenous production of hydroxytyrosol boosted the activity of the EADS and reduced the cytosolic contents of DA, DOPAL, and DOPAC in PC12 cells. Likewise, the overexpression of *ADH6* immediately converted DOPAL into hydroxytyrosol, leaving a very small amount of the precursor funnel through the production of DOPAC. The production of hydroxytyrosol in the PC12+syn^+^+sep^−^+ADH6^+^ cells was also confirmed by comparing the chromatogram of authentic standard of hydroxytyrosol and the sample (Supplementary Figure S4)

Taken together, this is first report on the endogenous production of hydroxytyrosol from DOPAL (an endogenous neurotoxin) by overexpressing a heterologous *ADH6* gene from *S. cerevisiae,* with which we analyzed the synergistic neuroprotective effect of synapsin-1 and septin-5 on PC12 cells against oxidative stress induced by salsolinol. The conversion of DOPAL into hydroxytyrosol not only prevents its accumulation in DAergic neurons, but also support the EADS of PC12 cells to relieve the oxidative stress induced by salsolinol.

## 3. Materials and Methods 

### 3.1. Plasmid Construction, Cell Line Construction, and Cell Culture

The rat pheochromocytoma PC12 cell line (ATCC^®^ CRL-1721™, *Rattus norvegicus*) was grown in Dulbecco’s modified Eagle’s medium (DMEM) medium with 10% fetal bovine serum (FBS) and 5% horse serum for 2–3 days. Afterward, PC12 cells were differentiated for 4–6 days by growing in DMEM medium containing 1% horse serum, 1% penicillin–streptomycin, and NGF (50 ng/ml) [84,85], and then transfected with pcDNA3.1(-)synapsin-1^+^, pcDNA3.1(-)septin-5^+^, pcDNA3.1(-)septin-5^−^, or pcDNA3.1(-)synapsin-1^+^+septin-5^−^ vectors at 80% confluency using Lipofectamine 2000 (Thermo Scientific, Waltham, MA, USA) for construction of the PC12+syn^+^, PC12+sep^+^, PC12+sep^−^, and PC12+syn^+^+sep^−^ cell lines, respectively (Supplementary Figure S1). The PC12+syn^+^+sep^−^+ADH6^+^ cell line was constructed by co-transfecting the pBROAD3-mcs+ADH6^+^ and pcDNA3.1(-)synapsin-1^+^+septin-5^−^ vectors in the PC12 cell using Lipofectamine 2000 (Supplementary Figure S1). The plasmids pcDNA3.1(-) and pBROAD3-mcs were purchased from Thermo Scientific, Waltham, MA, USA and InvivoGene, CA, USA. The cell lines produced in this study through genetic manipulation are listed in Supplementary Table S1. After 24 h, 500 ug/mL geneticin (Thermo Scientific, Waltham, MA, USA) was added for 15 days in the culture medium of each cell line, and drug-resistant single cell clones were further selected in the 96-well plate. All cell lines were grown in DMEM (Gibco, NY, USA) with 10% FBS (Gibco, NY, USA), 5% horse serum, and 100 U/mL penicillin–streptomycin (Sigma-Aldrich Co., St. Louis, USA) with 5% CO_2_ and at 37 °C for all types of experiments. The PC12 cell line was also previously used to determine the neuroprotective effects of different compounds and proteins [36,86,87,88]. The primers used for the construction of plasmids are given in Supplementary Table S1. The Xba1 and Kpn1 restriction sites were added at the forward and reverse primers of the synapsin-1 and septin-5 primers. The amplified fragments of synapsin-1 and septin-5 were separately ligated in the pcDNA3.1(-) using T4 DNA ligase (Thermo Scientific, Waltham, MA, USA). The pcDNA3.1(-)synapsin-1^+^ was double-digested with HindIII and AflII to construct the plasmid expressing both synapsin-1 and septin-5 siRNA. The expression cassette SV40p–septin-5(siRNA)–DAT1t of septin-5 siRNA was constructed through overlap extension PCR, where the forward primer of SV40p possesses the restriction site of HindIII and the reverse primer overlaps the 42 bp of siRNA of septin-5, and the forward primer of DAT1t overlaps the 42 bp of siRNA of septin-5 and the reverse primer possesses the restriction site of AflII (Supplementary Table S1). The expression cassette SV40p–septin-5(siRNA)–DAT1t constructed via OE-PCR (overlap extension PCR) was double-digested and ligated in the double-digested pcDNA3.1(-)synapsin-1^+^. The total DNA sequence of siRNA of septin-5 was 42 bp, which was added to the reverse primer of SV40p and the forward primers of DAT1t to create the overlaps for OE-PCR. The siRNA AACAUUCAGAGUUGUUCACCGGUGAACAACUCUGAAUG UUGG for septin-5 knockdown was designed using an online tool [89]. The primers used for ligation of ADH6 in the pBROAD3-mcs+ADH6^+^ were designed using the NEBuilder Assembly Tool (Supplementary Table S1). The restriction enzymes Xba1, Kpn1, HindIII, and AflII were purchased from Thermo Scientific, Waltham, MA, USA. The genes synapsin-1 (accession no. NM_001110780) and septin-5 (accession no. NM_213614.2) were amplified from cDNA extracted from *Mus musculus. ADH6* gene (accession no. NC_001145) was amplified from the genomic DNA of *Saccharomyces cerevisiae. ADH6* (*S. cerevisiae*) was codon optimized using the online tool JCAT for successful expression in PC12 cells and synthesized from Sangon Biotech Co. Ltd., Shanghai, China. The oligonucleotides used in this study were also synthesized from Sangon Biotech Co. Ltd., Shanghai, China.

### 3.2. IC_50_ of Salsolinol and MTS Assay

For the determination of salsolinol’s IC_50_ (Santa Cruz, CA, USA), doses of 0, 50, 100, 150, 200, 250, 300, 350, 400, 450, 500, and 550, µM were used and the cell viability was estimated through MTS assay (Supplementary Figure S2). The MILLIPORE Scepter hand-held automated cell counter (Merck & Co., USA) was used for cell counting. Cells were seeded in a 96-well plate at a density of 1 × 10^5^per well. After different administrations of salsolinol (≥96% pure), the MTS, along with PMS, was added to the culture medium, as recommended by the manufacturer (Sigma-Aldrich Co., St. Louis, USA). The plate was covered with aluminum foil and incubated at 37 °C with 5% CO_2_ for 2 h. A microplate reader, citation™ 5 (BioTek, Inc., VT, USA), was used to read the absorbance at 490 nm. Cell viability was calculated by using the following formula: cell sustainability (%) = OD (the experimental group) − Blank/OD (control) − Blank × 100%. In all experiments, except for PC12 cells, all cell lines were treated with an IC_50_ value 400 µM of salsolinol for 24 h to induce oxidative stress (Supplementary Figure S2).The MTS kit and related reagents were purchased from Sigma-Aldrich Co., St. Louis, USA. In this study, salsolinol was used as an exogenous neurotoxin to induce the oxidative stress in each cell line before performing each assay. Salsolinol is an endogenously synthesized neurotoxin that inflicts a neurotoxic effect on DAergic neurons by inhibiting the mitochondrial electron transport chain and producing oxidative stress [84]. The PC12 cells were pretreated with a 10, 50, or 100 µM dose of liquiritin, liquiritigenin, isoliquiritigenin, naringenin, hydroxytyrosol, p-Coumaric acid, cinnamic acid, or tyrosol for 4, 8, or 12 h to determine their dose-dependent neuroprotective effect against a 24-h post-incubation effect of the salsolinol. The optimum dose of each antioxidant was used for the optimum time period to determine their neuroprotective effect on the PC12 cells. Oxidative stress was induced for each experiment in all cell lines (except for PC12 cells) by using 400 µM of salsolinol (Supplementary Figure S2).

### 3.3. Western Blotting 

The cultured cells of PC12+syn^+^, PC12+sep^−^, PC12+syn^+^+ sep^−^, and PC12 cell lines were washed twice with pre-cold PBS, harvested, and then lysed with RIPA extraction buffer on ice for 30 min. Protease and phosphatase inhibitors (Roche, Basel, Switzerland) were added into the RIPA before its use. The protein concentration was determined by using the Bradford method. The Bradford assay reagents were purchased from Sigma-Aldrich Co., USA. Then, 5 µg/µL protein (50 µg in total/sample) was separated on 10% SDS-PAGE, and separated protein bands were transferred to 0.22 μm PVDF (Polyvinylidene difluoride) membrane (Millipore, Billerica, MA, USA). After blocking the membrane with 5% skim milk for 1.5 h at room temperature in TBST (Sigma-Aldrich Co., St. Louis, USA), it was incubated overnight with primary antibodies against β-actin (1:2000, ProteinTech, IL, USA), SEPT5 Rabbit (1:2000, ProteinTech, Illinois, USA), and synapsin-1 (D12G5) XP Rabbit mAb (1:1000, Cell Signaling, Boston, MA, USA) at 4 °C. The membrane was washed with TBST buffer for 50 min (10 min for each) and incubated with horseradish peroxidase-conjugated secondary antibody (Santa Cruz, CA, USA) for 1 h at room temperature. The bands were washed several times and developed with enhanced chemiluminescence’s reagents (Thermo Scientific, Waltham, MA, USA). After exposure to X-ray, the denseness of the bands was analyzed by the Bio-Rad imaging system Quantity One^®^ (Bio-Rad, CA, USA).

### 3.4. Assays for the Measurement of Biochemical Parameters

For the measurement of the biochemical parameters, the PC12, PoTwS, PC12+syn^+^, PC12+sep^−^, PC12+syn^+^+sep^−^, PC12+hydroxytyrosol, PC12+syn^+^+sep^−^+hydroxytyrosol, and PC12+syn^+^+sep^−^+ADH6^+^ cell lines were first grown in DMEM up to 80% confluency, treated with their selective treatment, and then old DMEM media were changed with fresh DMEM media and incubated with 400 µM salsolinol for 24 h afterward. Next, the cells were washed twice with precooled PBS and collected for lysis with lysis buffer provided by the manufacturers with each kit. The kits for analysis of oxidative stress were purchased from Abcam, Shanghai, China. Assays, i.e., MDA, H_2_O_2_, CAT, GSH, SOD, and GPx, were performed to analyze the oxidative stress level in each cell line constructed in this study (Supplementary Table S3). The kit used for the analysis of MDA was ab233471, Lipid Peroxidation Assay Kit, (Abcam, Shanghai, China); H_2_O_2_ was ab102500, Hydrogen Peroxide Assay Kit, (Abcam, Shanghai, China); CAT was ab83464, Catalase Activity Assay Kit (Abcam, Shanghai, China); GSH was ab239709 GSH+GSSG/GSH Assay Kit (Abcam, Shanghai, China); SOD was ab65354, Superoxide Dismutase Activity Assay kit (Abcam, Shanghai, China); and GPx was ab102530, Glutathione Peroxidase Assay Kit (Abcam, Shanghai, China). All experiments were performed by following the instructions of the manufacturer given in the manual with each kit. The MDA, H_2_O_2_, CAT, GSH, SOD, and GPx assays were performed with the PC12, PoTwS, PC12+hydroxytyrosol, PC12+syn^+^+sep^−^+hydroxytyrosol, and PC12+syn^+^+sep^−^+ADH6^+^ cell lines. Meanwhile, the PC12+syn^+^ and PC12+sep^−^ cell lines were only used to perform the MDA and H_2_O_2_ assays.

The difference between the means was considered significant at * *p* < 0.05 and ** *p* < 0.01, measured using the ANOVA test. 

### 3.5. Total RNA Extraction and qRT-PCR Analysis 

The PC12, PoTwS, PC12+hydroxytyrosol, PC12+syn^+^+sep^−^+hydroxytyrosol, and PC12+syn^+^+sep^−^+ADH6^+^ lines were separately grown for 3 days to achieve 80% confluency (Supplementary Table S3). The growth media of each cell line was removed, and 0.3–0.4 mL of TRIzol™ Reagent per 1 × 10^5^ – 1 × 10^7^ cells was directly added to the culture dish to lyse the cells (Invitrogen, Carlsbad, CA, US). The RNA extraction protocol was performed according to the manufacturer’s instructions. DNA contamination from RNA samples was excluded by adding 90 μL DNase I incubation buffer plus 10 μL DNase I into the RNA-containing reaction tubes; afterward, the reaction tubes were stored at 15 °C for 15 min. The concentration of total RNA in each sample was reckoned with the Nanodrop ND-1000 spectrophotometer (Thermo Scientific, Waltham, MA, USA) and the cDNA template was synthesized from 10,000 ng of total RNA using a Transcriptor First Strand cDNA Synthesis Kit (Roche, Basel, Switzerland). Real-time qPCR reactions were performed on the LightCycler 480 real-time System (Roche, Basel, Switzerland) and the reaction conditions were set as recommended by the SYBR Premix Ex TaqTM manual (Takara Biomedical Technology Co., Ltd. Beijing, China). The relative quantity of each gene was determined using the housekeeping gene glyceraldehyde-3-phosphate dehydrogenase (Gapdhs) as a reference, and data were analyzed with the Light Cycler Software (v.1.5). All assays were performed in triplicate and reactions without reverse transcriptase were used as negative controls. The primers for qRT-PCR analysis were designed with Beacon Designer™ 8.21 Free Edition software (PREMIER Biosoft, USA) (Supplementary Table S2).

### 3.6. HPLC Analysis of DA, DOPAL, DOPAC, and Hydroxytyrosol

PC12, PoTwS, PC12+hydroxytyrosol, PC12+syn^+^+sep^−^+hydroxytyrosol and PC12+syn^+^+sep^−^+ADH6^+^ cells were separately grown for 3 days in a T75 flask containing 5 ml DMEM up to 80% confluency. After that, PC12, PoTwS, PC12+hydroxytyrosol, and PC12+syn^+^+sep^−^+hydroxytyrosol and PC12+syn^+^+sep^−^+ADH6^+^ cells were harvested for analysis of DA, DOPAL, and DOPAC. The cell extract of PC12+syn^+^+sep^−^+ADH6^+^ was also used for the analysis of hydroxytyrosol. Cells were scraped from the flask and sonicated for 90 s (2 s on and 1 s off), and the supernatant was used for the extraction of DA, DOPAL, DOPAC, and hydroxytyrosol with an equal amount of ethyl acetate. The samples and standards were quantitatively analyzed by HPLC (Dionex Ultimate 3000). The HPLC analysis was carried out using Zorbax SB-C18 column (2.1 × 50 mm, 1.8 µm) and an Ultimate 3000 Photodiode Array Detector (Thermo Scientific, Waltham, MA, USA). The HPLC analysis program for DA, DOPAL, DOPAC, and hydroxytyrosol was set as flow rate, 0.5 mL min^−1^; solvent A, 100% acetonitrile; solvent B, 0.6% water containing formic acid. The peak of DA, DOPAL, DOPAC, and hydroxytyrosol in the respective samples was identified by comparing their LC spectra with authentic standards of DA (CAS. No. 62-31-7 and ≥98% pure), DOPAL (CAS. No.102-32-9 and ≥98% pure), DOPAC (CAS No. 102-32-9 and ≥98% pure), and hydroxytyrosol (CAS No. 10597-60-1 and ≥98% pure), respectively, purchased from Sigma-Aldrich Co., St. Louis, USA.

## 4. Conclusion

In this study, hydroxytyrosol was characterized as a strong neuroprotective agent compared to liquiritin, liquiritigenin, isoliquiritigenin, naringenin, p-Coumaric acid, cinnamic acid, and tyrosol. The overexpression of synapsin-1 and downregulation of septin-5 in PC12 cells also demonstrated a neuroprotective effect, perhaps due to their crucial role in the exocytosis and endocytosis of synaptic vesicles. DOPAL, an endogenous neurotoxin, is produced from the catabolism of DA, which can accumulate in DAergic neurons due to the oxidative stress produced during the onset of Parkinson’s disease. *ADH6* was overexpressed in the PC12 cells for endogenous conversion of DOPAL into hydroxytyrosol. Oxidative stress induced by salsolinol in the PC12+syn^+^+sep^+^+ADH6^+^ cells was efficiently relieved by endogenously-produced hydroxytyrosol by inducing the transcription of catalase, glutathione reductase, superoxide dismutase, and glutathione peroxidase. The endogenous production of hydroxytyrosol and the genetic manipulation of synaptic vesicle proteins also synergistically protected the PC12 cells from oxidative stress, perhaps through adopting different underlying pathways.

In a prospective study, *ADH6* gene will be expressed in an animal model using the AAV vector for the production of hydroxytyrosol from DOPAL; then, its effect on the EADS and the level of DOPAL in the animal model will be determined. Similarly, the expression of synaptic vesicle proteins will also be manipulated for the determination of their synergistic neuroprotective effect, along with endogenously-produced hydroxytyrosol on the animal model. This approach can bring about a breakthrough in the prevention of neurodegenerative disorders (e.g., PD) through disrupting the oxidative stress-mediated cascade of events.

## Supplementary Materials

The following are available online at https://www.mdpi.com/1420-3049/25/7/1715/s1, Figure S1: Design of plasmids used to construct the different cell lines, Table S1:. List of primers used for amplification and ligation of synapsin-1, septin-5, siRNA of septin-5 in pcDNA3.1(-) and ADH6 in pBROAD3-mcs. Figure S2: Determination of the IC50 of salsolinol for PC12 cells. Figure S3a,b,c: (a,b) Western blotting analysis of constructed cell lines. (c) Grey-scale analysis for quantification of synaptic vesicle proteins. Table S2: List of primers used for qPCR analysis. Figure S4a,b,c,d: HPLC analysis of different cell lines for detection and quantification of DA, DOPAL, DOPAC and Hydroxytyrosol. Table S3: List of cell lines constructed in this study and their description.
