# Supplementary material for: Synergistic Neuroprotective Effect of Endogenously-Produced Hydroxytyrosol and Synaptic Vesicle Proteins on Pheochromocytoma Cell Line against Salsolinol
Source: Molecules. 2020 Apr 8;25(7):1715. doi: 10.3390/molecules25071715 (PMC7181248; doi:10.3390/molecules25071715)
Supplement: Supplementary file 1 [file molecules-25-01715-s001.pdf]

Supplementary Materials

1. Scheme of plasmids used for construction of cell lines in this study.

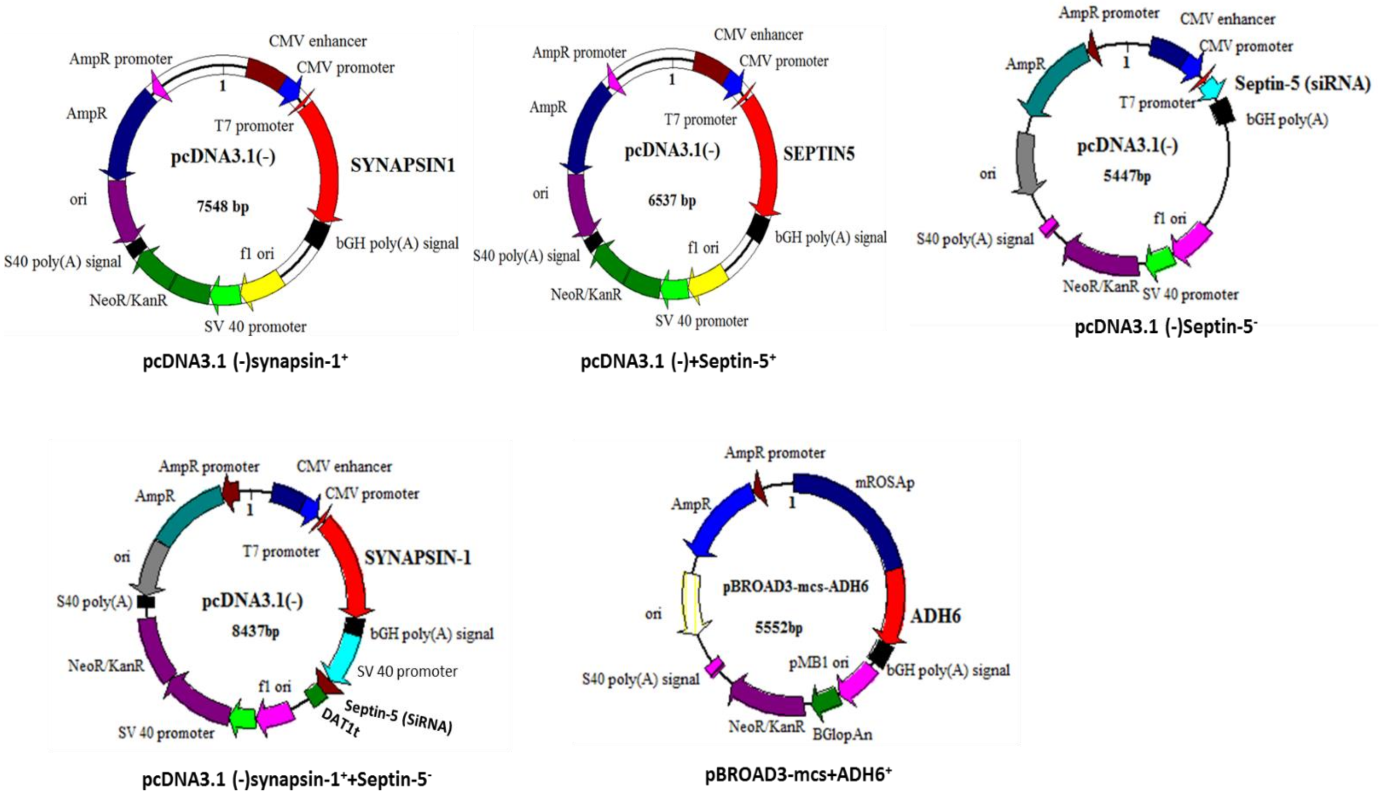

Figure S1. Plasmids used for construction of cell lines in this study.

Table S1. List of primers used for amplification and ligation of synapsin-1, septin-5, siRNA of septin-5 in pcDNA3.1(-) and ADH6 in pBROAD3-mcs.

Primers	Sequence
Septin-5-F	GCTCTAGAGC AGCCACC ATGAGCACAG GACTGCGGTA CAAAAGCAAG
Septin-5-R	GGGGTACCCCTCAGTGGTGATGGTGATGATGCTGGTCTTGCATTTGCTGCTTCATCTT
Synapsin-1F	GCTCTAGAGC AGCCACC ATGAACTACC TGCGGCGCCG
Synapsin-1-R	GGGGTACCCCTCAGTGGTGATGGTGATGATGGTCGGAGAAGAGGCTGGCGAAAGAC
ADH6-F	gacatgtagatctaagcttgATGTCTTATCCTGAGAAATTTG
ADH6-R	gcgagcttctagatggccagCTAGTCTGAAAATTCTTTGTC
SV40p-F	AAGCTT GTGTGTCAGT TAGGGTGTGG
SV40p-R	CCAACATTCAGAGTTGTTCACCGGTGAACAACTCTGAATGTTTTTGCAAAAGCCTAGGC CTC
DAT1-F	AACATTCAGAGTTGTTCACCGGTGAACAACTCTGAATGTTGGCGGTAAGCTCCCCAATG GGT
DAT1-R	CTTAAGTGTGAGGTGGCCCAGCTGGC

2. IC_{50} determination of salsolinol

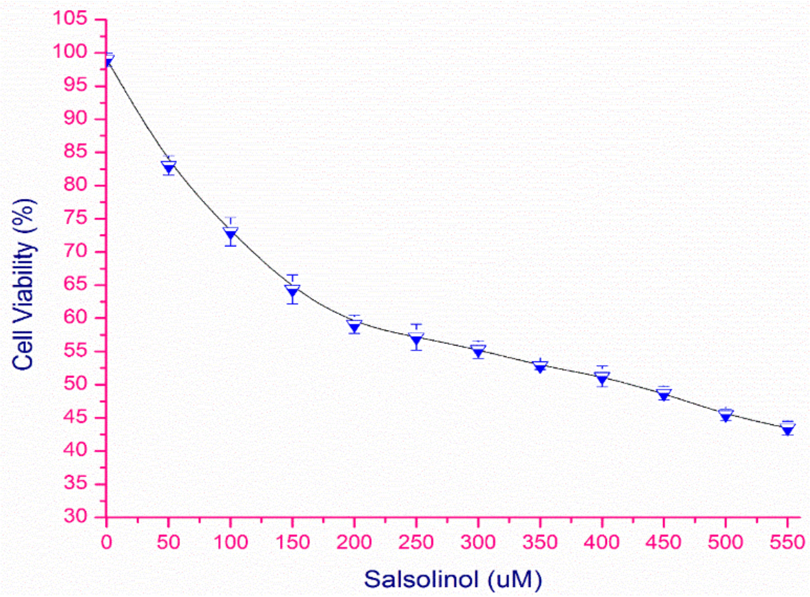

Figure S2. Determination of the IC_{50} of salsolinol for PC12 cells. A dose dependent decrease in cell viability of PC12 cells was observed using salsolinol. It was found that salsolinol killed 51.24 ± 1.58% of PC12 cells at 400 uM dose. Error bars represent the standard deviation of biological triplicate.

3. Expression level analysis of synaptic vesicle proteins in constructed cell lines

The overexpression of synapsin-1 and downregulation of septin-5 in respective cell line was determined using western blotting (Supplementary Figure 3a, b).

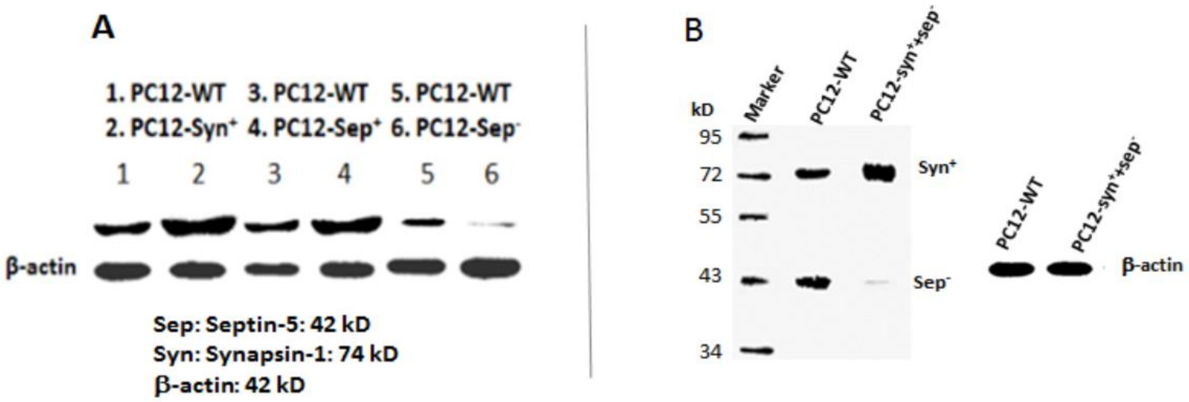

Figure S3a, b. Western blotting of constructed cell lines. Western blotting results showed that the expression of synapsin-1 was increased in the PC12+syn^{+} by up to 32.25% and expression of septin-5 was downregulated in the PC12+Sep^{-} by up to 75.25 % compared to PC12-WT (PC12) (Supplementary Figure 3c). The PC12+syn^{+}+sep^{-} cell line displayed a 34.34 % increase in the expression of synapsin-1 and the downregulation of septin-5 by 77 % compared to PC12-WT.

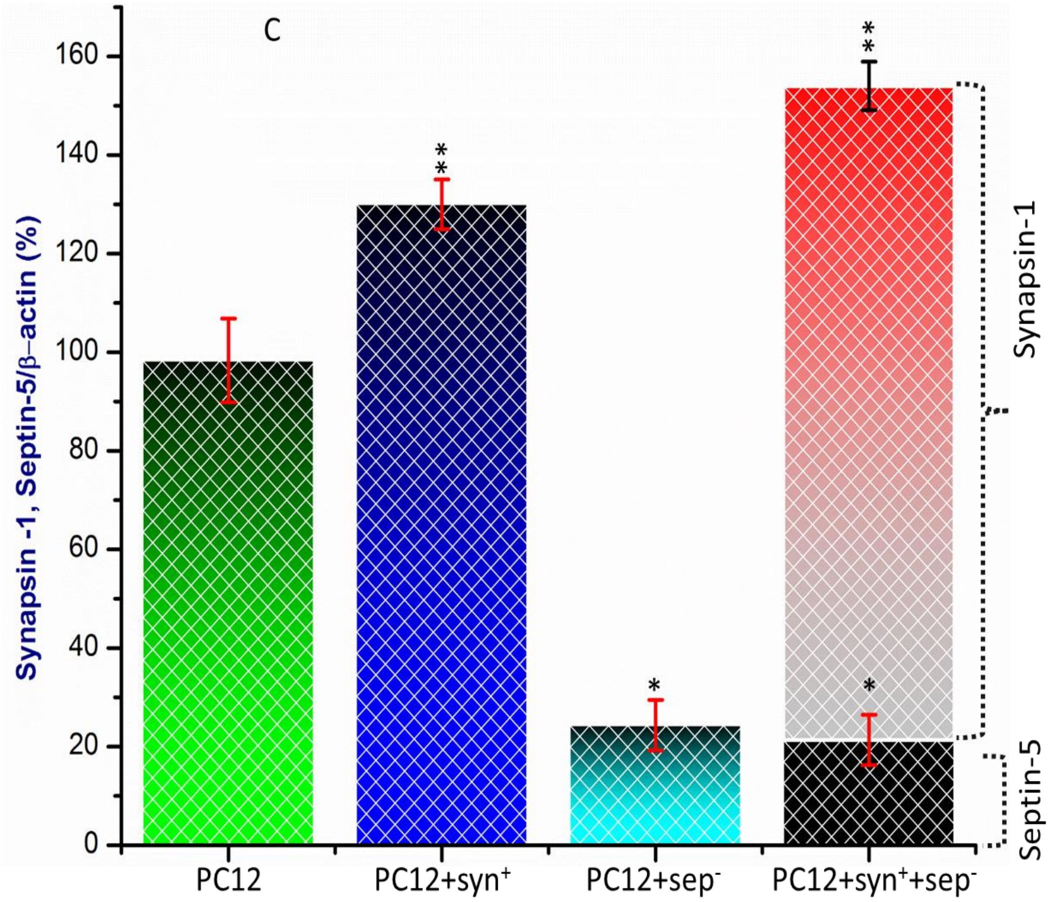

Figure S3c. Grey-scale analysis of overexpression of synapsin-1 and downregulation of septin-5 compared with β-actin. It demonstrated that expression of synapsin-1 was increased in PC12+syn$^+$ cells compared to the PC12 cells (PC12-WT). While expression of septin-5 was downregulated in PC12+sep- cells compared to the PC12 cells. And, PC12+syn$^+$+sep$^-$ cell displayed an increased expression level of synapsin-1 and decreased expression of septin-5 compared to PC12 cells. The description of each cell line is given in the Supplementary Table 3. Data are mean and S.E. values from three independent experiments (n = 3). *$p<0.05$, and **$p < 0.01$ relative to PC12 cells.

Table S2. List of primers used for qPCR analysis.

Primers	Sequence
**catalase-F**	GTCACCCACGATATTACCAGAT
**catalase-R**	GAAACAACATGGCATCCCTGAT
**Superoxide dismutase-F**	: TGACCTGCCTTACGACTATG
**Superoxide dismutase-R**	GATAGCCTCCAGCAACTCTC
**glutathione peroxidase-F**	GGACTACACCGAAATGAATGAT
**glutathione peroxidase-R**	CCTCGCACTTCTCAAACAAT
**glutathione reductase-F**	ATTTAACCAAGTCCCACATCGAAG
**glutathione reductase-R**	TCCAGCTGAAAGAACCCATC
**Gapdhs-F**	ATGGGTGTGAATGAGAAGGA
**Gapdhs-R**	AGTGGAAGATGGGATGATGT

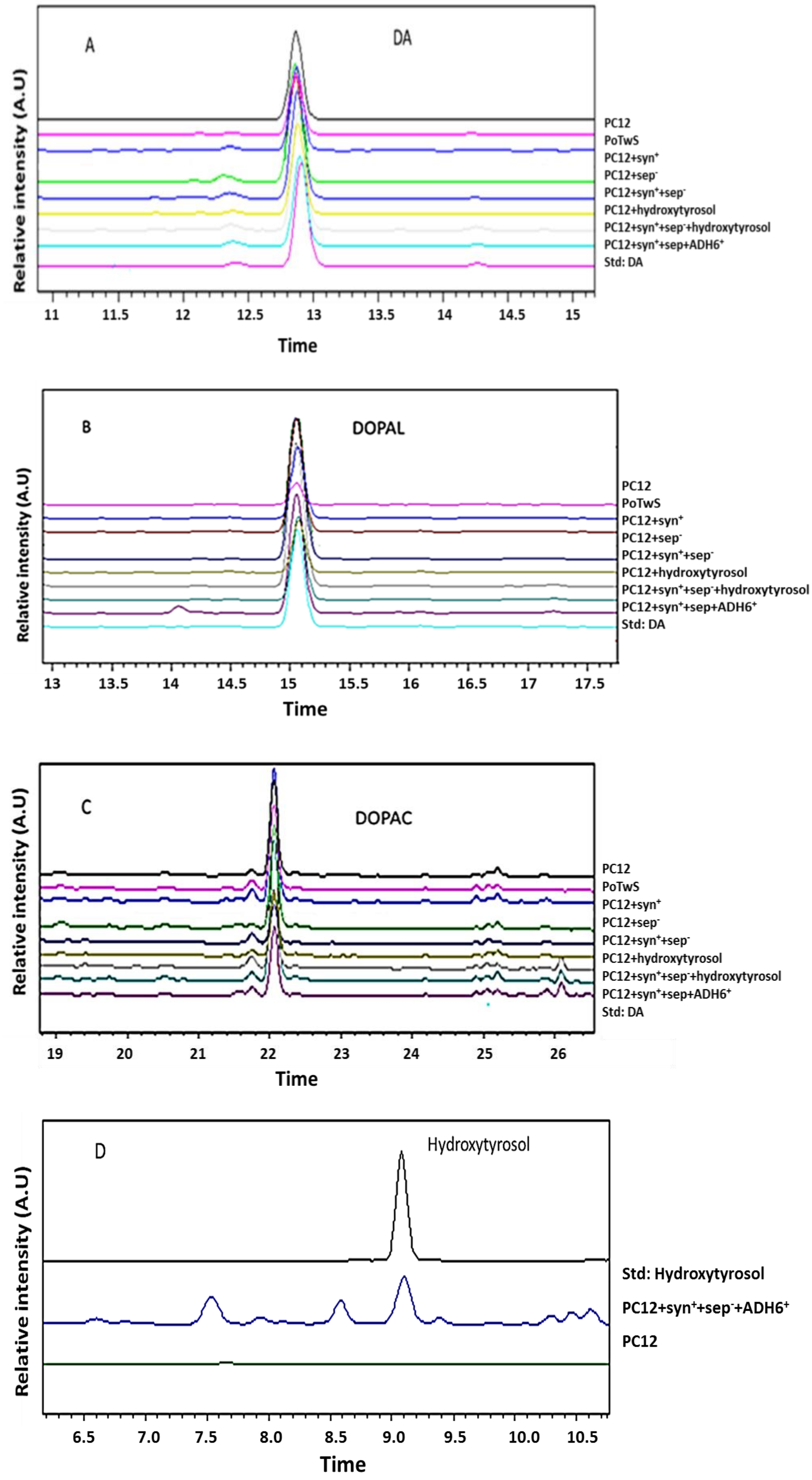

Figure S4. HPLC analysis of different cell lines for detection and quantification of DA, DOPAL, DOPAC and Hydroxytyrosol.

Table S3. List of cell lines made in this study and their description.

#	CELL LINES	DESCRIPTION OF CELL LINE
1.	**PC12 cells**	Control PC12 cell without any manipulation
2.	**PC12+salsolinol (PowTS)**	PC12 cell treated with salsolinol (only) for 24 hours prior to each assay
3.	**PC12+syn^{+}**	PC12 cells overexpressing synapsin-1 and post-incubated for 24 hours with salsolinol prior to each experiment
4.	**PC12+sep^{+}**	PC12 cells overexpressing septin-5 and incubated for 24 hours with salsolinol prior to each assay
5.	**PC12+sep^{-}**	PC12 cells with septin-5 knockdown and incubated for 24 hours with salsolinol prior to each assay
6.	**PC12+syn^{+}+sep^{-}**	PC12 cells overexpressing synapsin-1 and septin-5 knockdown and incubated for 24 hours with salsolinol prior to each assay
7.	**PC12+hydroxytyrosol**	PC12 cells pretreated with hydroxytyrosol and incubated for 24 hours with salsolinol prior to each assay
8.	**PC12+syn^{+}+sep^{-}**	PC12 cells overexpressing synapsin-1 and septin-5 knockdown and incubated for 24 hours with salsolinol prior to each assay
9.	**PC12+syn^{+}+sep^{-} +hydroxytyrosol**	PC12 cells overexpressing synapsin-1 , septin-5 knockdown and pretreated with hydroxytyrosol and then incubated for 24 hours with salsolinol prior to each assay
10.	**PC12+syn^{+}+sep^{-} +ADH^{+}**	PC12 cells overexpressing synapsin-1, and alcohol dehydrogenase 6 and septin-5 knockdown and then incubated for 24 hours with salsolinol prior to each assay.
